# Salmonella

**DOI:** 10.3201/eid2612.ET2612

**Published:** 2020-12

**Authors:** Daniel F. M. Monte, Fábio P. Sellera

**Affiliations:** University of São Paulo, São Paulo, Brazil

**Keywords:** etymologia, Salmonella, bacteria, salmonellosis, serotypes, typhoid fever, flagellar antigens, Daniel Elmer Salmon, zoonoses

## *Salmonella* [sal¢¢mo-nel¢ә]

Named in honor of Daniel Elmer Salmon, an American veterinary pathologist, *Salmonella* (Figure) is a genus of motile, gram-negative bacillus, nonspore-forming, aerobic to facultatively anaerobic bacteria of the family *Enterobacteriaceae*. In 1880, Karl Joseph Eberth was the first to observe *Salmonella* from specimens of patients with typhoid fever (from the Greek *typhōdes* [like smoke; delirious]), which was formerly called *Eberthella typhosa* in his tribute. In 1884, Georg Gaffky successfully isolated this bacillus (later described as *Salmonella* Typhi) from patients with typhoid fever, confirming Eberth’s findings. Shortly afterward, Salmon and his assistant Theobald Smith, an American bacteriologist, isolated *Salmonella* Choleraesuis from swine, incorrectly assuming that this germ was the causative agent of hog cholera. Later, Joseph Lignières, a French bacteriologist, proposed the genus name *Salmonella* in recognition of Salmon’s efforts. 

**Figure:**
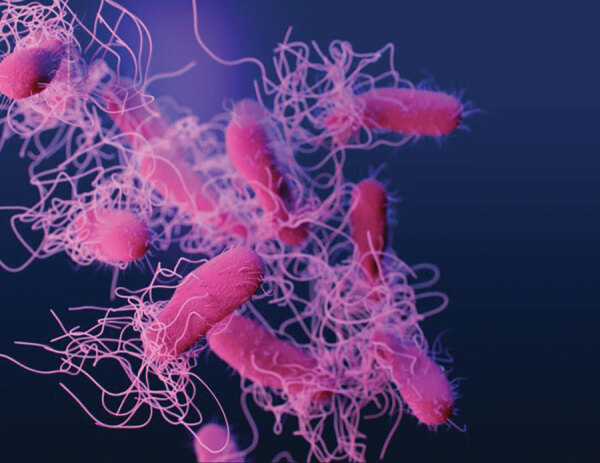
Drug-resistant, nontyphoidal, Salmonella sp. bacteria showing numerous flagella. Taken from Antibiotic Resistance Threats in the United States, 2019 (AR Threats Report); Centers for Disease Control and Prevention. Illustration: James Archer/ CDC, 2019.

With a complicated taxonomy, the genus *Salmonella* is currently classified into 2 species (*S. enterica* and *S. bongori*), encompassing 2,659 serotypes based on somatic O and H flagellar antigens as specified in the Kauffmann–White–Le Minor scheme. *S. enterica* is divided into 6 subspecies: *enterica*, *salamae*, *arizonae*, *diarizonae*, *houtenae*, and *indica*. Arguably, this zoonotic pathogen remains one of the most pressing global concerns. It causes a spectrum of diseases in several hosts, and there is much to be learned and deciphered about its continuous evolution.
